# DNA replication stress: oncogenes in the spotlight

**DOI:** 10.1590/1678-4685-GMB-2019-0138

**Published:** 2019-12-13

**Authors:** Luiza M. F. Primo, Leonardo K. Teixeira

**Affiliations:** 1 Group of Cell Cycle Control, Program of Immunology and Tumor Biology. Brazilian National Cancer Institute (INCA), Rio de Janeiro, RJ, Brazil

**Keywords:** Cancer, cell cycle, DNA replication, oncogene, replication stress

## Abstract

Precise replication of genetic material is essential to maintain genome stability. DNA replication is a tightly regulated process that ensues faithful copies of DNA molecules to daughter cells during each cell cycle. Perturbation of DNA replication may compromise the transmission of genetic information, leading to DNA damage, mutations, and chromosomal rearrangements. DNA replication stress, also referred to as DNA replicative stress, is defined as the slowing or stalling of replication fork progression during DNA synthesis as a result of different insults. Oncogene activation, one hallmark of cancer, is able to disturb numerous cellular processes, including DNA replication. In fact, extensive work has indicated that oncogene-induced replication stress is an important source of genomic instability in human carcinogenesis. In this review, we focus on main oncogenes that induce DNA replication stress, such as RAS, MYC, Cyclin E, MDM2, and BCL-2 among others, and the molecular mechanisms by which these oncogenes interfere with normal DNA replication and promote genomic instability.

## DNA replication

Eukaryotic chromosomes are precisely replicated once each cell cycle to ensure genome stability. The process of DNA replication is conserved among different organisms and is tightly controlled by the sequential assembly of various proteins onto DNA replication origins (ORIs), followed by the concerted synthesis of nascent DNA strands. In mammalian cells, ORIs are generally characterized as nucleosome-free, GC-rich genomic regions where DNA replication starts. Multiple protein complexes function in a coordinated fashion to recognize ORIs, unwind double-strand DNA, and perform DNA synthesis. Through the renowned semiconservative process, DNA replication is performed by different DNA polymerases, which require single-strand DNA (ssDNA) templates to build complementary DNA molecules: one continuous strand in the same direction as the replication fork progression (the leading strand) and one discontinuous strand in the opposite direction through the generation of short Okazaki fragments (the lagging strand) (Masai *et al.*, 2010; Leonard and Méchali, 2013; O’Donnell *et al.*, 2013).

To ensure one round of DNA replication per cell cycle, cells precisely control the execution of two temporally separated steps before the onset of DNA synthesis: origin licensing and origin firing. During late mitosis and early G1 phase, when cells experience low cyclin-dependent kinase (CDK) environments, origin licensing is accomplished by the sequential assembly of protein complexes onto ORIs. Origin licensing occurs through the loading of origin recognition complex subunits 1-6 (ORC1-6), cell division cycle 6 (CDC6) protein, and chromatin licensing and DNA replication factor 1 (CDT1), followed by recruitment of DNA helicase minichromosome maintenance complex components 2-7 (MCM2-7) to form pre-replication complexes (pre-RC). At the pre-RC stage, the helicase complex is inactive and unable to unwind the double-strand DNA molecule. Once origin licensing is completed, cells activate several mechanisms to inhibit a new round of origin licensing within the same cell cycle, and therefore prevent DNA rereplication, such as inhibitory phosphorylation and ubiquitin-mediated degradation of pre-RC components among other mechanisms (Masai *et al.*, 2010; McIntosh and Blow, 2012; Siddiqui *et al.*, 2013).

The second critical step before the onset of DNA replication occurs during the G1/S phase transition, when additional proteins are assembled onto chromatin to establish pre-initiation complexes (pre-IC). Contrary to origin licensing, origin activation requires high CDK activity and is triggered by the concerted action of CDC7 and CDK2 protein kinases, which associate with the regulatory subunits DBF4 and Cyclin E/A, respectively. These S phase kinases phosphorylate several replication factors during pre-IC assembly and activate the DNA helicase complex through facilitating the recruitment of CDC45 and GINS complex to form the CMG complex (CDC45-MCM-GINS). Activation of the CMG helicase then unwinds the double-strand DNA and further allows the recruitment of other replication factors, such as replication factor C (RFC), replication protein A (RPA), the sliding clamp proliferating cell nuclear antigen (PCNA), and multiple DNA polymerases, all essential for initiation of DNA synthesis and replication fork movement (replisome formation). It is important to point out that the vast majority of licensed origins along the genome are not activated during normal S phases and remain on hold as backup (dormant) ORIs to serve in specific physiological situations, such as DNA replication stress. Furthermore, the subset of activated origins in a given cell varies at each cell cycle and also differs among different cells, underscoring the importance of ORI activation dynamics and flexibility in DNA replication and other cellular functions (Masai *et al.*, 2010; Méchali, 2010; Tanaka and Araki, 2013; Fragkos *et al.*, 2015).

Once ORIs are activated, DNA synthesis is triggered in S phase by replisomes (large replication machineries) at thousands of chromosomal sites with two replication forks progressing in opposite directions, a process known as origin firing. In close association with several replication factors (such as TopBP1, RecQL4, Treslin, and MCM10), the CMG complex moves along the DNA molecule, generating transient ssDNA and replication forks. DNA polymerases then catalyze the incorporation of deoxyribonucleoside triphosphates (dNTPs) to build two DNA strands that are complementary to the parental DNA molecule. DNA replication priming (synthesis initiation of a new DNA strand) is accomplished by the DNA polymerase alpha-primase complex, which synthesizes RNA/DNA hybrid primers, while replication elongation is primarily performed by DNA polymerase epsilon at the leading strand and DNA polymerase delta at the lagging strand through generation of 100-200 nucleotides long Okazaki fragments. In normal cell cycles, origin firing occurs at approximately 30-50,000 sites along the 3 billion base pairs of human chromosomes and DNA replication forks travel roughly at 1-2 Kb per minute, ensuring completion of chromosomal replication in about 8 hours during S phase. Importantly, DNA polymerases exonucleolytic proofreading activities and sophisticated DNA repair mechanisms work in coordination to generate high fidelity DNA molecules and preserve genome integrity (Johansson and Dixon, 2013; Lujan *et al.*, 2016; Burgers and Kunkel, 2017).

## Mechanisms of oncogene-induced replication stress

DNA replication stress, also known as DNA replicative stress, is characterized by the slowing or stalling of replication fork progression during DNA synthesis, which may lead to replication fork collapse and DNA damage. If not resolved by replication checkpoint mechanisms, persistent replication stress may cause mutations, copy number alterations (CNAs, amplifications and deletions), and chromosomal rearrangements (Zeman and Cimprich, 2014; Gaillard *et al.*, 2015; Técher *et al.*, 2017). In normal conditions, one of the main consequences of DNA replication stress is the activation of the DNA damage response (DDR) pathway, which is primarily triggered by the generation of ssDNA upon fork stalling. ssDNA creates a platform for recruitment and activation of several proteins, such as RPA, Ataxia Telangiectasia and Rad3-related (ATR), and Checkpoint Kinase 1 (CHK1), which subsequently recruit and activate numerous substrates to inhibit cell cycle progression, stabilize stalled replication forks, and promote DNA replication restart. Importantly, activation of the DDR pathway has been proposed to function as an inducible barrier during early stages of tumorigenesis, leading to cell cycle arrest, cell death or senescence. DDR deficiency compromises cellular checkpoints, causes DNA damage, and genomic instability, and is associated with cancer susceptibility (Bartkova *et al.*, 2005, 2006; Gorgoulis *et al.*, 2005; Di Micco *et al.*, 2006; Halazonetis *et al.*, 2008). The mechanisms of DDR activation upon DNA replication stress have been extensively reviewed in the literature and are beyond the scope of this article (Sirbu and Cortez, 2013; Blackford and Jackson, 2017; Saldivar *et al.*, 2017; Toledo *et al.*, 2017). In this section, we briefly discuss the main mechanisms of oncogene-induced replication stress.

**Figure 1:**
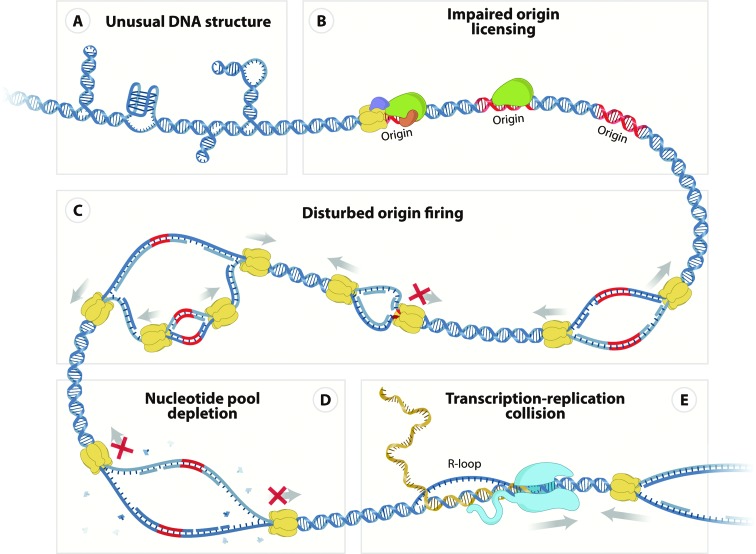
Molecular mechanisms of DNA replication stress. A) Unusual DNA secondary structures may be formed at certain genomic regions, such as centromeres, telomeres, and fragile sites, and represent natural obstacles to replication fork progression. Stem-loop (left and right) and G-quadruplex (middle) structures are represented. B) Impaired origin licensing may compromise the formation of active replication origins and DNA replication. Normal (left), impaired (middle), and absence of (right) pre-RC formation are represented. C) Disturbed origin firing may interfere with DNA replication and replication fork progression. Normal (right), asymmetric (middle), and repetitive (left) origin firing are represented. D) Uncontrolled S phase entry in the presence of nucleotide pool depletion may impair DNA replication and prevent replication fork progression. E) Collisions between replication and transcription machineries may impair DNA replication fork progression through generation of DNA topological stress and formation of persistent R-loops, RNA-DNA hybrid molecules. A-E) DNA molecule (blue strand), DNA origin of replication (Origin, red strand), ORC complex (green), CDC6 protein (orange), CDT1 protein (purple), MCM complex (yellow), RNA polymerase (blue), and messenger RNA (yellow strand) are represented. DNA polymerases and replisomes are omitted for simplicity. Grey arrows represent progression of replication or transcription machineries and red crosses represent stalled replication forks.

Oncogene activation, one established hallmark of cancer, is able to directly interfere with normal DNA replication and represents an important source of replication stress and genomic instability. Oncogene activation causes replication stress through different mechanisms, such as impairment of origin licensing and/or origin firing, nucleotide pool depletion, and interference between DNA replication and transcription machineries (Figure 1).

Unusual DNA structures may be formed at specific genomic regions during certain cellular processes that generate ssDNA, such as DNA replication, transcription, and DNA repair (Bochman *et al.*, 2012; Kaushal and Freudenreich, 2019). Formation of DNA secondary structures normally occurs at repetitive nucleotide sequences and represents one important obstacle to replisome progression (Figure 1A). Several different alternative DNA structures, such as stem-loop and G-quadruplex (G4), may be formed at AT- and GC-rich regions, and can lead to increased DNA torsional stress, replication fork stalling, double-strand DNA breaks (DSBs), and chromosome fragility (Ozeri-Galai *et al.*, 2011; Chambers *et al.*, 2015; Tubbs *et al.*, 2018). In fact, oncogene activation may interfere with normal replication and pose further risk to genomic regions with these DNA secondary structures, which have been mapped to breakpoint hotspots and regions with CNAs in human cancers (Tsantoulis *et al.*, 2008; Beroukhim *et al.*, 2010; Bignell *et al.*, 2010). The consequences of unusual DNA structures to chromosome replication and fragility will be further discussed in the next section.

Origin licensing is the initial step of DNA replication and must be precisely coordinated through the cell cycle to allow appropriate origin firing in S phase (McIntosh and Blow, 2012). As discussed before, the vast majority of licensed origins constitute backup (dormant) ORIs that are not activated during normal S phase. Accordingly, it has been shown that depletion of pre-RC proteins does not interfere with DNA replication in unperturbed cells (Ge *et al.*, 2007; Ibarra *et al.*, 2008). However, under conditions of challenged DNA replication, deficient assembly of pre-RC proteins reduces the number of functional ORIs, impairing DNA replication and causing replication stress (Figure 1B). Indeed, substantial interference with ORC2, CDT1 or MCM2 loading onto chromatin arrests cells in G1 and prevents S phase progression, most likely because of insufficient origin licensing (Shreeram *et al.*, 2002; Machida *et al.*, 2005). Oncogene activation has also been shown to directly inhibit the loading of MCM complex proteins onto chromatin, resulting in impaired origin firing and fork progression (Ekholm-Reed *et al.*, 2004; Bartkova *et al.*, 2006).

Following origin licensing, coordinated origin firing is also essential for accurate DNA replication (Fragkos *et al.*, 2015). Reduced or asymmetric origin firing may force replication forks to travel for longer distances along the genome, increasing the chances of replication fork collapse (Figure 1C). Impaired ORI activation may also decrease replication fork velocity, allowing cells to enter into mitosis with incompletely replicated genomes. In fact, oncogene activation has been shown to inhibit origin firing and lead to unscheduled DNA replication (Frum *et al.*, 2014). On the other hand, oncogene activation may also induce replication stress through increased origin firing (Vaziri *et al.*, 2003). Multiple ORI activation at specific genomic sites can lead to a second round of DNA replication within one cell cycle, a process known as DNA rereplication (Figure 1C). Indeed, overexpression of pre-RC components, such as CDT1 and CDC6, increases origin firing, induces DNA rereplication, and has been observed in different human cancers (Vaziri *et al.*, 2003; Di Micco *et al.*, 2006; Liontos *et al.*, 2007).

Nucleotides are essential components of nucleic acids and are necessary for DNA replication (Lane and Fan, 2015). The nucleotide biosynthesis pathway must be precisely coordinated within cells to maintain normal levels of deoxyribonucleotides and ensure normal DNA replication. Oncogene activation may induce uncontrolled S phase entry with insufficient nucleotide pools (Figure 1D). In fact, it has been shown that oncogene overexpression is able to induce increased cell proliferation with exhausted dNTP levels, leading to replication fork stalling and DSBs (Bester *et al.*, 2011). Also, oncogene activation may directly interfere with nucleotide biosynthesis, causing dNTP pool depletion and premature termination of replication forks (Aird *et al.*, 2013; Xie *et al.*, 2013).

Finally, DNA replication stress may be also caused by collisions between replication and transcription machineries. These conflicts usually occur at genomic sites that encode large genes (> 800 Kb), which require more than one round of cell cycle to complete transcription and therefore are transcriptionally active during S phase (Helmrich *et al.*, 2011). Transcription-replication collisions may lead to DNA topological constraints and persistent accumulation of R-loops, RNA-DNA hybrid molecules generated during transcription (Helmrich *et al.*, 2013). If not resolved, these structures may cause replication fork stalling, DNA damage, and chromosome breakage (Figure 1E). Another potential consequence of unresolved transcription-replication collisions is the formation of unusual DNA replication intermediates, such as reversed replication forks (Neelsen and Lopes, 2015). Indeed, it has been shown that oncogene activation induces conflicts between replication and transcription machineries due to increased transcriptional activity and R-loop formation, leading to replication stress and DNA damage (Jones *et al.*, 2013; Kotsantis *et al.*, 2016). The molecular mechanisms of oncogene-induced replication stress have been discussed in detail by others (Hills and Diffley, 2014; Macheret and Halazonetis, 2015; Kotsantis *et al.*, 2018).

## Genomic regions susceptible to replication stress

Certain genomic regions present intrinsic difficulties to accomplish DNA synthesis upon perturbed DNA replication. Among these regions, common fragile sites (CFS) have been defined as chromosomal loci that are prone to breaks and/or gaps in situations of replication stress. These sites are usually characterized by AT-rich sequences and ORI paucity, are located at late-replicating domains, and contain large isolated genes (Debatisse *et al.*, 2012; Ozeri-Galai *et al.*, 2012; Glover *et al.*, 2017). Repetitive AT sequences may lead to formation of DNA secondary structures, which impose natural obstacles to replication fork progression (Ozeri-Galai *et al.*, 2011). Lack of ORI activation forces distant converging replication forks to travel for long distances to finish DNA synthesis, increasing the risk of incomplete DNA replication (Letessier *et al.*, 2011). Genomic regions that replicate late in S phase also present an increased likelihood of incomplete DNA replication because there might not be enough time to complete DNA synthesis within S phase (Le Beau *et al.*, 1998). Finally, as discussed before, chromosomal loci with large, actively transcribed genes are more susceptible to collisions between replication and transcription machineries, also contributing to CFS instability (Helmrich *et al.*, 2011).

CFS strongly correlate with recurrent deletions in a broad spectrum of human tumors (Tsantoulis *et al.*, 2008; Beroukhim *et al.*, 2010; Bignell *et al.*, 2010). FRA3B and FRA16D are the two most frequently affected CFS in human cancers, including breast, lung, colon, esophageal, and renal carcinomas (Durkin and Glover, 2007). FRA3B is located at 3p14.2 and overlaps with the 1.5 Mb-long *Fragile Histidine Triad* (*FHIT*) tumor suppressor gene, which is involved in nucleotide metabolism (Saldivar and Park, 2019). FRA3B instability is caused by a paucity of replication initiation events at the central region of this fragile site, as well as transcription-replication collisions due to extended transcription of the large *FHIT* gene (Helmrich *et al.*, 2011; Letessier *et al.*, 2011). FRA16D is located at 16q23 and overlaps with the 1.1 Mb-long *WW Domain Containing Oxidoreductase* (*WWOX*) tumor suppressor gene, which is involved in apoptotic and DDR pathways (Hussain *et al.*, 2019). Similar to FRA3B, FRA16D fragility is also associated with scarcity of initiation events and transcription-replication collisions at the large *WWOX* gene (Helmrich *et al.*, 2011; Letessier *et al.*, 2011). In addition to FRA3B and FRA16D, other CFS, such as FRA6E, FRA9E, and FRA7G, present intrinsic vulnerabilities, are susceptible to major genomic losses, and have been shown to contribute to human carcinogenesis (Durkin and Glover, 2007; Glover *et al.*, 2017).

Although late-replicating genomic regions are susceptible to chromosomal fragility, early-replicating fragile sites (ERFS) have also been shown to be vulnerable to replication stress and DNA damage (Mortusewicz *et al.*, 2013). Unlike CFS, ERFS are characterized by GC-rich sequences, repetitive elements, increased ORI density, and highly transcribed gene clusters. Upon S phase entry, these genomic regions show high ORI activity close to transcriptionally active genes, leading to replication fork stalling, DSBs, and chromosome rearrangements (Barlow *et al.*, 2013). It is therefore likely that ERFS instability is induced by increased conflicts between replication and transcription machineries. Importantly, many ERFS overlap with recurrent CNAs at genomic regions implicated in the development of human diffuse large B cell lymphomas (Barlow *et al.*, 2013).

Besides CFS and ERFS, other genomic regions are also inherently difficult to replicate and susceptible to replication stress. Two clear examples are telomeres and centromeres, which are both heterochromatic regions enriched in repetitive sequences. These chromosomal regions are prone to formation of complex DNA secondary structures, such as stem-loops, G4 structures, and DNA catenanes, which can interfere with replication fork progression and contribute to chromosome fragility (Martínez and Blasco, 2015; Bloom and Costanzo, 2017; Higa *et al.*, 2017; Black and Giunta, 2018). Sophisticated protein complexes regulate telomere and centromere stability and function. Disruption of several telomere- and centromere-binding proteins has been shown to impair resolution of DNA secondary structures, induce replication fork stalling, and cause fragility at these loci (Martínez *et al.*, 2009; Sfeir *et al.*, 2009; Aze *et al.*, 2016; Giunta and Funabiki, 2017). In addition, oncogene activation has been demonstrated to induce chromosome breaks at centromeres and generate aberrant structures at telomeres in response to replication stress (Suram *et al.*, 2012; Miron *et al.*, 2015).

## Oncogenes in the spotlight

DNA replication must be precisely regulated during cell cycle in order to ensure genome stability. An extensive body of work has clearly demonstrated that oncogene activation induces replication stress at susceptible genomic sites through different molecular mechanisms (Figure 1). In the following sections, we discuss in detail the effects of the main human oncogenes that have been shown to cause DNA replication stress.

### RAS

Oncogenic RAS has been closely related to DNA replication stress. The RAS family is composed of three proto-oncogenes (*K-*, *H-*, and *N-RAS*) that function as small GTPase signal transducers. RAS proteins are essential components of a network that communicate cell surface receptors with intracellular proteins to regulate cellular growth, survival, and metabolism among other functions. Under physiological conditions, these G proteins are activated upon GTP binding and then activate downstream effectors that regulate several mitogenic pathways, including the RAF/MEK/ERK and the PI3K/AKT pathways. Somatic mutations in *RAS* cause its constitutive activation and the subsequent stimulation of effectors that promote cell proliferation, apoptosis suppression, and metabolic reprogramming. *RAS* alterations are frequently observed in human cancers, specifically *K-RAS* mutations, which are found in approximately 40% of colorectal cancers and 20% of lung adenocarcinomas (Karnoub and Weinberg, 2008; Pylayeva-Gupta *et al.*, 2011).

Sustained mitogenic stimulation by oncogenic RAS (H-RASV12) directly impinges on DNA replication and causes replication stress through several mechanisms (Table 1). In a groundbreaking work, Di Micco and collaborators have shown that oncogenic RAS induces replication stress by increasing origin firing and generating asymmetric replication forks (Di Micco *et al.*, 2006). It is possible that the increased origin firing reflects on DNA rereplication induced by the licensing factor CDC6, as it has been shown that RAS overexpression upregulates the levels of CDC6. It has also been demonstrated that oncogenic RAS interferes with cellular dNTP levels by downregulating the ribonucleotide reductase subunit M2 (RRM2), causing dNTP pool depletion and premature termination of replication forks (Aird *et al.*, 2013). Together with others, these observations have contributed to the notion that oncogene-induced replication stress leads to a robust DDR activation and an irreversible cell cycle arrest, a phenotype known as oncogene-induced senescence (OIS) (Bartkova *et al.*, 2006; Di Micco *et al.*, 2006, 2007; Mallette *et al.*, 2007). In fact, oncogene-induced DDR activation, followed by cell death or senescence, has been proposed to function as an inducible barrier against human tumorigenesis (Bartkova *et al.*, 2005, 2006; Gorgoulis *et al.*, 2005; Di Micco *et al.*, 2006; Halazonetis *et al.*, 2008).

**Table 1:** Mechanisms of DNA replication stress induced by different oncogenes.

Oncogene	Mechanism of replication stress	Reference
RAS	Increased origin firing	Di Micco *et al*., 2006
	Impaired fork progression	Di Micco *et al*., 2006; Maya-Mendoza *et al*., 2015
	Nucleotide pool depletion	Aird *et al*., 2013
	Transcription-replication collision	Kotsantis *et al*., 2016
MYC	Disturbed origin firing	Dominguez-Sola *et al*., 2007; Srinivasan *et al*., 2013; Macheret and Halazonetis, 2018
	Impaired fork progression	Srinivasan *et al*., 2013; Maya-Mendoza *et al*., 2015
CCNE1	Unusual DNA structure	Teixeira *et al*., 2015
	Decreased origin licensing	Ekholm-Reed *et al*., 2004
	Disturbed origin firing	Liberal *et al*., 2012; Jones *et al*., 2013; Macheret and Halazonetis, 2018
	Impaired fork progression	Bartkova *et al*., 2006; Bester *et al*., 2011; Costantino *et al*., 2014
	Replication fork reversal	Neelsen *et al*., 2013
	Nucleotide pool depletion	Bester *et al*., 2011
	Transcription-replication collision	Jones *et al*., 2013; Macheret and Halazonetis, 2018
CDC6	Increased origin firing	Vaziri *et al*., 2003; Sideridou *et al*., 2011
	Transcription-replication collision	Huang *et al*., 2016; Komseli *et al*., 2018
CDC25	Increased origin firing	Cangi *et al*., 2008
	Replication fork reversal	Neelsen *et al*., 2013
MDM2	Decreased origin firing	Frum *et al*., 2014
	Impaired fork progression	Klusmann *et al*., 2016
BCL-2	Nucleotide pool depletion	Xie *et al*., 2013

Oncogenic RAS may also induce replication stress as a consequence of oxidative stress. Initial expression of oncogenic RAS causes hyperproliferation and increases the velocity of replication forks. However, overexpression of RAS for longer periods of time causes cellular metabolic changes and reduces fork progression (Di Micco *et al.*, 2006; Maya-Mendoza *et al.*, 2015). It has been demonstrated that RAS-induced senescence is triggered by increased production of reactive oxygen species (ROS) (Irani *et al.*, 1997; Lee *et al.*, 1999), which lead to nucleotide oxidation as well as H_2_O_2_ generation (Rai *et al.*, 2011; Weyemi *et al.*, 2012). Alleviation of these oxidative insults by different approaches prevents DNA damage and cellular senescence. Therefore, it is possible that oxidative stress contributes to RAS-induced replication stress through accumulation of oxidized DNA precursors and generation of DSBs (Leikam *et al.*, 2008; Maya-Mendoza *et al.*, 2015).

Another mechanism of replication stress induced by RAS is increased global transcription. RAS proteins promote cellular proliferation through upregulation of general transcription factors that are able to stimulate RNA synthesis (Pylayeva-Gupta *et al.*, 2011). Indeed, it has been shown that oncogenic RAS leads to elevated expression of the TBP transcription factor (TATA-box binding protein) and increased transcriptional activity. Elevated RNA synthesis causes replication fork slowing and DNA damage through collisions between replication and transcription machineries and subsequent formation of R-loops (Kotsantis *et al.*, 2016). Interestingly, TBP overexpression alone is able to increase transcription and cause replication stress and DNA damage, recapitulating the effects of oncogenic RAS.

Other mechanisms may also contribute to RAS-induced replication stress. One possibility is the interference with DNA repair. It has been shown that oncogenic RAS causes dissociation of BRCA1 protein from chromatin, compromising DNA repair and leading to DNA damage (Tu *et al.*, 2011). Inactivation of BRCA1 protein renders cells susceptible to accumulation of secondary mutations and potentially cancer development. In light of the numerous insults caused by oncogenic RAS in DNA replication, it is reasonable to speculate that RAS-induced replication stress may result in genomic instability. In fact, RAS activation has been shown to induce chromosome abnormalities, such as acentric fragments, deletions, and double minute chromosomes (Denko *et al.*, 1994; Guerra *et al.*, 2003), replication fork stalling at telomeres, leading to telomere attrition and aberrant telomeric structures (Suram *et al.*, 2012), and genomic alterations at CFS relevant to human carcinogenesis (Tsantoulis *et al.*, 2008; Miron *et al.*, 2015).

### MYC

The MYC family of transcription factors in composed of the three members: C-, L-, and N-MYC. MYC proteins are effectors of several signaling transduction pathways and control a variety of cellular functions, including cell growth, proliferation, differentiation, and apoptosis. As a transcription factor, MYC primarily mediates its functions through dimerization with MAX and binding DNA regulatory elements to regulate an array of gene transcription programs. Additionally, MYC proteins also play nontranscriptional roles in cellular physiology. Activation of oncogenic MYC usually occurs through gene amplification, chromosomal rearrangement or loss of upstream MYC regulators, leading to sustained levels of MYC and interference with essential cellular processes. In fact, deregulation of c-MYC expression is observed in more than half of human cancers and oncogenic MYC has been associated with aggressive breast, prostate, and colon cancers, as well as Burkitt lymphoma (Dang, 2012; Dominguez-Sola and Gautier, 2014; Rohban and Campaner, 2015).

MYC-induced replication stress is triggered by different molecular mechanisms and generates DNA damage and genomic instability during carcinogenesis (Table 1). Initial evidence indicated that MYC-induced genomic instability was associated with oxidative stress. c-MYC overexpression causes alterations in cellular metabolism, including increased production of ROS, which correlates with DNA damage (Vafa *et al.*, 2002). However, in contrast to RAS, oncogenic MYC causes replication stress before induction of cellular metabolic changes (Maya-Mendoza *et al.*, 2015). In fact, several studies have subsequently demonstrated that MYC activation leads to DNA damage and genomic instability through direct impairment of DNA replication dynamics (Karlsson *et al.*, 2003; Ray *et al.*, 2006; Dominguez-Sola *et al.*, 2007; Sankar *et al.*, 2009; Srinivasan *et al.*, 2013).

The main mechanism of MYC-induced replication stress is through interference with origin firing. It has been demonstrated that MYC localizes to ORIs and physically interacts with pre-RC components during origin licensing, such ORCs, CDC6, CDT1, and MCMs (Dominguez-Sola *et al.*, 2007). MYC also participates in ORI activation by increasing the recruitment of CDC45 to chromatin, a replication factor that is essential for initiation of DNA replication (Dominguez-Sola *et al.*, 2007; Srinivasan *et al.*, 2013). In accordance, MYC depletion decreases the number of active ORIs, while MYC overexpression leads to increased and premature origin firing. Once deregulated, oncogenic MYC leads to ORI hyperactivation, replication fork asymmetry and stalling, and eventually DNA damage (Dominguez-Sola *et al.*, 2007; Srinivasan *et al.*, 2013; Maya-Mendoza *et al.*, 2015). Importantly, these effects of MYC on origin firing have been shown to be independent of its transcriptional activity. Similar to the well-characterized effect of oncogenic Cyclin E1 in origin firing (discussed below), MYC overexpression also induces changes in genomic location of ORI activation from intergenic to intragenic regions with high transcriptional activity (Macheret and Halazonetis, 2018). Considering that MYC is a transcription factor and that its overexpression upregulates transcription and increases origin firing, it is reasonable to speculate that oncogenic MYC also causes replication stress by generating collisions between replication and transcription machineries. However, this potential mechanism of MYC-mediated replication stress remains to be demonstrated.

An indirect mechanism for MYC-induced replication stress is through activation of Cyclin E/CDK2 complex. It has been widely demonstrated that oncogenic MYC promotes cell cycle progression and increases Cyclin E/CDK2 activity, which may be achieved by induction of *CCND2* gene expression, inactivation of CDK inhibitor p27^Kip1^ or stimulation of E2F transcription factor-dependent genes, among other mechanisms (Bretones *et al.*, 2015). The specific consequences of increased Cyclin E/CDK2 activity to replication stress are discussed in the following section.

In contrast to the above, MYC proteins can intriguingly counteract replication stress through several mechanisms. As mentioned earlier, MYC transcription factors induce expression of numerous genes involved in cellular proliferation and DNA replication, including the nucleotide biosynthesis pathway (Liu *et al.*, 2008; Mannava *et al.*, 2008). Interestingly, c-MYC expression increases purine and pyrimidine metabolism and provides sufficient nucleotide pools to rescue replication stress induced by high rates of DNA synthesis upon disruption of the RB-E2F pathway (Bester *et al.*, 2011). Furthermore, MYC proteins directly upregulate the expression of certain enzymes involved in DNA replication, such as the WRN helicase (Werner syndrome), a protein involved in the resolution of unusual replication intermediates, and the MRN nuclease (MRE11/RAD50/NBS1), a complex responsible for DSB repair and restart of collapsed replication forks (Grandori *et al.*, 2003; Robinson *et al.*, 2009; Petroni *et al.*, 2016). Upregulation of WRN helicase and MRN nuclease constitute safeguard mechanisms to protect cells from replication stress and DNA damage upon MYC expression.

Oncogenic MYC is frequently associated with human tumorigenesis. As discussed above, MYC overexpression induces replication stress and DSBs, which may be eventually associated with genomic instability. In fact, it has been shown that oncogenic MYC causes chromosomal aberrations, such as deletions, amplifications, and translocations, aneuploidy, and telomeric fusions (Felsher and Bishop, 1999; Karlsson *et al.*, 2003; Louis *et al.*, 2005). Oncogenic MYC has also been shown to induce fragility at specific genomic sites, such as CFS and ERFS (Barlow *et al.*, 2013).

### Cyclin E

Cyclin E is one of the prototypical oncogenes that induce replication stress. The Cyclin E family is composed of two proteins, Cyclin E1 and E2 (CCNE1 and CCNE2), which share similar gene sequences and cellular functions. Normally, Cyclin E protein levels peak at the G1/S transition and are completely degraded by the end of S phase. In association with CDK2, Cyclin E controls DNA replication through phosphorylation of multiple proteins, such as the RB tumor suppressor and the DNA replication factors CDT1, CDC6, and Treslin. RB phosphorylation leads to release of E2F transcription factors, which induce the expression of various genes required for DNA replication, while phosphorylation of DNA replication factors is essential for origin licensing and origin firing. Therefore, it is not surprising that oncogenic activation of Cyclin E interferes with cell cycle progression and DNA replication, causing replication stress and genomic instability. *CCNE1* amplification, overexpression or impaired protein degradation has been observed in premalignant lesions and cancers, such as breast and lung tumors, and leukemias (Hwang and Clurman, 2005; Siu *et al.*, 2012; Teixeira and Reed, 2017).

Many different mechanisms have been shown to contribute to Cyclin E-induced replication stress (Table 1). Unscheduled levels of Cyclin E1 directly interfere with pre-RC formation during late mitosis and early G1 phase, specifically with the recruitment of the helicase subunits MCM2, MCM4, and MCM7 to chromatin (Ekholm-Reed *et al.*, 2004). Inefficient assembly of pre-RC prevents appropriate origin licensing and compromises origin firing and DNA synthesis initiation. Indeed, it has been observed that Cyclin E1 overexpression results in either decreased (Liberal *et al.*, 2012) or increased origin firing (Jones *et al.*, 2013) in different models. Besides interference with origin licensing and origin firing, Cyclin E overexpression also impairs replication fork progression. High levels of Cyclin E1 cause premature termination of replication forks, fork collapse, and DSBs (Bartkova *et al.*, 2005, 2006). It has been shown that replication fork collapse induced by Cyclin E can be repaired by the homologous recombination pathway break-induced replication (BIR), further leading to copy number alterations and genomic instability (Costantino *et al.*, 2014). It is important to note that replication stress induced by Cyclin E is dependent on CDK2, as high levels of CDK2 activity are sufficient to impair replication fork progression and cause DNA damage (Hughes *et al.*, 2013).

Another primary mechanism for Cyclin E-induced replication stress is reduction of nucleotide pools. Through disruption of the RB/E2F pathway, Cyclin E1 overexpression enforces cell hyperproliferation with insufficient nucleotide levels, interfering with replication fork progression and causing DSBs (Bester *et al.*, 2011). Interestingly, cellular supplementation with exogenous nucleosides or induction of nucleotide metabolism through c-MYC expression are able to attenuate replication stress and DNA damage induced by Cyclin E1 overexpression.

Cyclin E-induced replication stress is also caused by transcription-replication collisions, which can lead to DNA topological stress and formation of persistent R-loops. Inhibition of transcription elongation has been shown to alleviate replication stress and reduce DNA damage caused by oncogenic Cyclin E1 (Jones *et al.*, 2013). Consistently, inhibition of replication initiation also restores normal levels of fork progression upon high levels of Cyclin E1. Together, these results indicate that oncogenic Cyclin E1 induces replication stress through generation of transcription-replication conflicts. One potential consequence of these encounters is the formation of DNA replication intermediates that are generated in response to topological stress, such as reversed replication forks. Indeed, high levels of Cyclin E1 induce the appearance of aberrant reversed forks (Neelsen *et al.*, 2013).

In a recent work, the human genome has been mapped respective to ORI distribution and replication timing under normal and high levels of Cyclin E1 (Macheret and Halazonetis, 2018). Under normal conditions, ORIs are predominantly activated in intergenic regions. Instead, overexpression of Cyclin E1 leads to shortened G1, rapid S phase entry, and novel origin firing in intragenic regions with high transcriptional activity. Excessive origin firing in protein-coding genes facilitates conflicts between transcription and replication machineries, generating replication fork collapse, DSBs, and chromosomal rearrangements (Macheret and Halazonetis, 2018).

As discussed before, intrinsic genomic characteristics may sensitize cells to replication stress upon oncogenic insults. In fact, Cyclin E1 deregulation allows cells to enter into mitosis with incomplete replication at specific genomic segments, resulting in mitotic aberrations, such as chromosome breaks and anaphase bridges, as well as CNAs (Teixeira *et al.*, 2015). Genomic fragility caused by Cyclin E1 overexpression shows several features of CFS, such as low origin density, late-replicating domains, very long genes, and DNA secondary structures (Miron *et al.*, 2015; Teixeira *et al.*, 2015; Teixeira and Reed, 2017). Accordingly, genomic breakpoints and rearrangements induced by Cyclin E1 overexpression *in vitro* are reflected in a large cohort of human cancers with *CCNE1* amplification (Zack *et al.*, 2013; Miron *et al.*, 2015; Teixeira *et al.*, 2015; Macheret and Halazonetis, 2018).

### CDC6

CDC6 is a DNA replication-licensing factor that is essential for pre-RC assembly during late mitosis and early G1. Specifically, CDC6 facilitates the loading of MCM helicase to ORIs and is also able to mediate the activation of cell cycle checkpoints and regulate gene transcription (Borlado and Méndez, 2008). Aberrant expression of CDC6 induces several oncogenic properties *in vitro*, such as DDR activation, cellular transformation, and genomic instability, as well as tumor growth *in vivo*. Furthermore, high levels of CDC6 have been observed in advanced stages of non-small cell lung carcinoma (NSCLC) and colon cancer (Bartkova *et al.*, 2006; Liontos *et al.*, 2007; Sideridou *et al.*, 2011).

As expected for a protein involved in pre-RC formation, unbalanced levels of CDC6 during cell cycle progression interfere with origin licensing and/or activation (Table 1). The initial evidence for CDC6-induced replication stress came from the observation that overexpression of CDC6, in cooperation with CDT1, promotes origin refiring and DNA rereplication in p53-deficient cells within a few hours of S phase, leading to amplification of large genomic segments and genomic instability (Vaziri *et al.*, 2003). Later on, oncogenic CDC6 was confirmed to increase ORI activation at specific genomic sites through chromatin displacement of the CTCF chromosome insulator (Sideridou *et al.*, 2011). Additionally, Bartkova and colleagues have shown that high levels of CDC6 induce RPA foci formation, an indicative of ssDNA that has been consistently associated with stalled replication forks (Bartkova *et al.*, 2006).

Besides increased origin firing and DNA rereplication, CDC6-induced replication stress can also occur through collision between replication and transcription machineries and formation of R-loops (Komseli *et al.*, 2018). Interestingly, R-loop formation caused by CDC6 overexpression is preferentially observed within the nucleoli, consistent with the fact that CDC6 is important for transcriptional regulation of the highly repetitive heterochromatic ribosomal DNA (rDNA) (Huang *et al.*, 2016). As a result of replication stress, CDC6 upregulation causes a number of structural and numerical chromosome aberrations in different models, with the majority of breakpoints located at CFS (Liontos *et al.*, 2007; Sideridou *et al.*, 2011; Komseli *et al.*, 2018). As discussed before, it is important to consider that CDC6 upregulation may be a consequence of RAS or Cyclin E1 oncogene activation, leading to DNA rereplication, DDR activation, and genomic instability (Mailand and Diffley, 2005; Di Micco *et al.*, 2006).

### Other oncogenes

Besides the well-characterized roles of RAS, MYC, Cyclin E1, and CDC6 oncoproteins in replication stress, several other oncogenes are also associated with this condition (Table 1). The CDC25 family of proteins is composed of three phosphatases (CDC25A, B, and C) that play critical roles in cell cycle progression and checkpoint control. At particular cell cycle stages and under certain conditions, CDC25 phosphatases directly dephosphorylate and activate CDKs to promote cell cycle transitions. Also, DDR activation triggers CDC25 degradation upon DNA damage, leading to CDK inactivation and cell cycle arrest in order to mediate DNA repair, cell death, or senescence. CDC25 oncogenic properties have been illustrated by cellular transformation, aneuploidy, and tumor formation *in vivo*, either in cooperation with oncogenic RAS or *RB1* loss (Boutros *et al.*, 2007). In agreement with its role as an oncogene, CDC25 overexpression has been documented in a variety of human cancers and correlated with disease aggressiveness and poor patient prognosis (Galaktionov *et al.*, 1995; Cangi *et al.*, 2000).

Initial overexpression of CDC25A causes unscheduled origin activation and DDR induction, while sustained levels of CDC25A leads to checkpoint disruption and chromosomal breaks (Mailand *et al.*, 2000; Bartkova *et al.*, 2005; Cangi *et al.*, 2008). Importantly, it has been shown that CDC25A overexpression slows down replication fork progression and induces reversed forks (Neelsen *et al.*, 2013). Besides CDC25A, other members of the CDC25 family also seem to be associated with replication stress, indicating a conserved function for these proteins in regulating cell cycle checkpoints and DDR activation. Increased levels of CDC25B or CDC25C interfere with DNA replication, leading to DNA damage, premature mitotic entry, and chromosomal aberrations (Varmeh and Manfredi, 2009; Bugler *et al.*, 2010). However, the molecular mechanisms for these events have not been completely elucidated.

One proto-oncogene that is essential for cell cycle/death control and has been associated with DNA replication stress is the mouse double minute 2 (MDM2) human protein. MDM2 directly interacts with the tumor suppressor p53 to regulate several cellular processes. MDM2 inactivates p53 transactivation domain, promotes its export from the nucleus to the cytoplasm, and induces p53 ubiquitin-mediated degradation. As a negative regulator of p53, it is not surprising that *MDM2* amplification and/or overexpression are frequently observed in human cancers, such as many subtypes of sarcomas as well as gliomas and leukemias (Karni-Schmidt *et al.*, 2016). It has been shown that MDM2 overexpression inhibits origin firing through activation of the intra S-phase checkpoint, causing unscheduled DNA replication (Frum *et al.*, 2014). Conversely, it has also been shown that p53 activation and subsequent MDM2 upregulation both enhance replication fork progression and increase replication fork processivity (Klusmann *et al.*, 2016). Although these findings appear conflicting, it is tempting to speculate that disruption of the p53/MDM2 axis in human cancers, either by *TP53* mutation or MDM2 overexpression, may interfere with origin firing and replication fork stability. The precise molecular mechanism by which MDM2 overexpression controls origin activation and causes replication stress remains to be determined.

B-cell lymphoma 2 (BCL-2) is another proto-oncogene involved in cell death regulation that has also been linked to replication stress. BCL-2 anti-apoptotic protein promotes cell survival primarily by coordinating protein interactions at several cellular compartments to control mitochondrial membrane permeability. Overexpression of BCL-2 inhibits cell death, facilitates the acquisition of genetic alterations during tumorigenesis, and is frequently observed in human malignancies, including follicular lymphoma, leukemia, and lung carcinoma (Delbridge *et al.*, 2016). Concerning the process of DNA replication, it has been shown that BCL-2 directly inhibits ribonuclease reductase (RNR) activity through binding and disruption of the RRM1/RRM2 complex formation (Xie *et al.*, 2013). BCL-2-induced RNR inhibition leads to decreased intracellular levels of dNTPs, slower progression of replication forks, and replication fork asymmetry, all classical features of replication stress.

Oncogenic alterations in the PI3K/AKT signaling pathway represent another insult frequently observed in human cancers. However, alterations in PIK3CA or AKT have not been unequivocally associated with replication stress to date. On the other hand, the PTEN tumor suppressor protein, which counterbalances the PI3K/AKT pathway in the cytoplasm, has been clearly linked to DNA replication, DNA repair, and genome stability in the nucleus (Brandmaier *et al.*, 2017; Lee *et al.*, 2018). Indeed, it has been shown that *PTEN* loss impairs replication fork progression and causes replication fork stalling during unperturbed conditions (He *et al.*, 2015). Under conditions of replication stress, PTEN is also essential for stability and recovery of stalled replication forks (Feng *et al.*, 2015; He *et al.*, 2015; Wang *et al.*, 2015). Several independent mechanisms have been proposed to explain the requirement for PTEN in protecting DNA replication forks. PTEN facilitates the recovery of stalled forks by directly recruiting RAD51 to chromatin, a recombinase that plays multiple roles in DNA replication and repair (He *et al.*, 2015). Additionally, upon replication stress, PTEN restricts replication fork progression through dephosphorylation of MCM2, potentially regulating MCM complex function (Feng *et al.*, 2015). Finally, PTEN has also been shown to protect replication forks through stabilization of the ssDNA-binding protein RPA1 in a phosphatase-independent manner (Wang *et al.*, 2015). Together, these studies indicate that PTEN disruption may lead to progressive accumulation of replication errors, DNA damage, and ultimately contribute to genomic instability in cancer.

## Conclusions and Perspectives

Normal DNA replication is essential to maintain genome stability in all living organisms. Perturbations in DNA replication may compromise transmission of genetic information to daughter cells, leading to DNA damage and mutations. In fact, increased frequency of DNA replication errors during stem cell divisions has been shown to be associated with higher cancer incidence in humans (Tomasetti and Vogelstein, 2015). In precancerous lesions, one important source of DNA replication errors is oncogene activation, which leads to sustained cellular proliferation and DNA replication stress. Elucidating the causes and consequences of oncogene-induced replication stress is therefore fundamental for better understanding human carcinogenesis.

An extensive body of work has shown that a number of oncogenic insults induce replication stress and genomic instability in human cells. Interestingly, distinct oncogenes, such as *H-RAS* and *CCNE1*, are able to generate unique genome fragility landscapes in the same cell type (Miron *et al.*, 2015). As discussed in previous sections, this can be explained by the fact that each oncogene induces replication stress through specific mechanisms. In addition, it is clear that one same replicative insult (either oncogenic or not) causes particular genomic alterations in distinct cell types, including fibroblasts, lymphocytes, and epithelial cells (Le Tallec *et al.*, 2011, 2013; Hosseini *et al.*, 2013; Miron *et al.*, 2015; Teixeira *et al.*, 2015). Specific genomic fragility among different cell types is possibly related to cell-type specific chromatin structure and organization, DNA replication timing, and transcriptional activity among other factors (Alabert and Groth, 2012; Sima and Gilbert, 2014; Santos-Pereira and Aguilera, 2015). Together, these observations indicate that replication stress induced by specific oncogenes can create unique repertoires of genomic alterations in different human cell types and cancers.

Replication stress has been considered a potential vulnerability of cancer cells and represents a promising target for cancer therapy. In cancer cells, replication stress may be largely attributed to constitutive oncogene activation. Indeed, multiple signs of oncogene-induced replication stress and consequent DDR pathway activation are frequently observed in precancerous lesions. Recent therapeutic approaches have focused on identifying synthetic lethal interactions between cancer-associated mutations and DNA replication vulnerabilities (Ubhi and Brown, 2019). It has been proposed that, under specific conditions of oncogenic activation, inhibition of DDR proteins induces extensive replication stress, irreversible DSBs, and subsequent cell death, leading to selective elimination of cancer cells. In fact, transformed cells and tumors showing replication stress induced by MYC, RAS, or Cyclin E1 oncoproteins are highly sensitive to ATR or CHK1 kinase inhibitors in different *in vitro* and *in vivo* models (Gilad *et al.*, 2010; Murga *et al.*, 2011; Toledo *et al.*, 2011; Schoppy *et al.*, 2012). Several combined therapies of traditional chemotherapeutic agents with DDR inhibitors are under investigation in clinical trials and have shown promising results to cancer patients. Some of the current challenges for improving the efficacy of replication stress-based therapies consist of identifying particular tumor types that are more likely to respond to specific treatments, determining optimal treatment strategy combinations, and establishing precise therapeutic doses and windows for intervention without generating adverse side effects. Over the coming years, the field of oncogene-induced replication stress will certainly experience further fundamental, exciting discoveries.
